# Targeting Metabolic Vulnerabilities to Combat Drug Resistance in Cancer Therapy

**DOI:** 10.3390/jpm15020050

**Published:** 2025-01-27

**Authors:** Taranatee Khan, Manojavan Nagarajan, Irene Kang, Chunjing Wu, Medhi Wangpaichitr

**Affiliations:** 1 Department of Veterans Affairs, Miami VA Healthcare System, Miami, FL 33125, USA; taranate@usc.edu (T.K.); mxn816@miami.edu (M.N.); kangi@usc.edu (I.K.); chunjing.wu@va.gov (C.W.); 2 Sylvester Comprehensive Cancer Center, University of Miami, Miami, FL 33136, USA; 3 South Florida VA Foundation for Research and Education, Miami, FL 33125, USA; 4 Department of Surgery, Division of Thoracic Surgery, University of Miami, Miami, FL 33136, USA

**Keywords:** cancer, drug resistance, tumor metabolism, immunometabolism, oxidative metabolism

## Abstract

Drug resistance remains a significant barrier to effective cancer therapy. Cancer cells evade treatment by reprogramming their metabolism, switching from glycolysis to oxidative phosphorylation (OXPHOS), and relying on alternative carbon sources such as glutamine. These adaptations not only enable tumor survival but also contribute to immune evasion through mechanisms such as reactive oxygen species (ROS) generation and the upregulation of immune checkpoint molecules like PD-L1. This review explores the potential of targeting metabolic weaknesses in drug-resistant cancers to enhance therapeutic efficacy. Key metabolic pathways involved in resistance, including glycolysis, glutamine metabolism, and the kynurenine pathway, are discussed. The combination of metabolic inhibitors with immune checkpoint inhibitors (ICIs), particularly anti-PD-1/PD-L1 therapies, represents a promising approach to overcoming both metabolic and immune evasion mechanisms. Clinical trials combining metabolic and immune therapies have shown early promise, but further research is needed to optimize treatment combinations and identify biomarkers for patient selection. In conclusion, targeting cancer metabolism in combination with immune checkpoint blockade offers a novel approach to overcoming drug resistance, providing a potential pathway to improved outcomes in cancer therapy. Future directions include personalized treatments based on tumor metabolic profiles and expanding research to other tumor types.

## 1. Introduction

Cancer treatment has undergone remarkable advancements over decades, with chemotherapy, targeted therapies, and immunotherapies significantly improving patient outcomes. Despite this progress, the emergence of drug resistance remains a major hurdle. We, along with other scientists, have demonstrated that cancer cells rewire their metabolic pathways to sustain proliferation and growth [1,2,3,4]. This metabolic reprogramming and immune evasion mechanisms make these resistant cancers therapeutically challenging.

Recent evidence highlights that targeting the metabolic dependencies of cancer cells could offer a promising avenue to overcome drug resistance. As cancer cells adapt their metabolism, they develop specific vulnerabilities that can be therapeutically exploited. After acquiring resistance to chemotherapy, cancer cells often become more reliant on mitochondrial oxidative phosphorylation (OXPHOS), offering a distinctive metabolic target.

This review explores the metabolic adaptations driving drug resistance in cancer, with a particular focus on the Warburg effect, oxidative phosphorylation, and the kynurenine pathway. Furthermore, it examines potential therapeutic strategies that leverage these metabolic weaknesses, including the combination of metabolic inhibitors with immune checkpoint blockade, representing a novel approach to overcoming drug resistance in cancer.

## 2. Cancer Metabolism and Its Role in Therapy Resistance

Cancer cells exhibit profound alterations in metabolism that distinguish them from normal cells. These metabolic changes support the rapid proliferation of cancer cells and contribute to their ability to evade apoptosis and develop resistance to therapy. The most well-known metabolic adaptation in cancer cells is the Warburg effect, first described by Otto Warburg, which refers to the preference of cancer cells to utilize glycolysis for energy production, even in the presence of oxygen [5,6]. This process, known as aerobic glycolysis, allows cancer cells to produce ATP and RNA more rapidly, though less efficiently, than through OXPHOS [7,8,9].

However, studies suggest that drug-resistant cancer cells, particularly in aggressive tumors (i.e., NSCLC, melanoma, and breast cancer), undergo further metabolic reprogramming that allows them to survive chemotherapy [10,11,12,13]. Rather than relying exclusively on glycolysis, resistant cells often shift back to OXPHOS, becoming more dependent on mitochondrial function to meet their energy needs [14,15,16]. We reported that the shift toward oxidative metabolism (OXMET) is particularly evident in cisplatin-resistant lung cancer cells, where increased mitochondrial activity leads to elevated levels of reactive oxygen species (ROS) [1,2,17].

The ROS produced during oxidative metabolism can have both damaging and signaling effects. Elevated ROS levels can induce oxidative stress, DNA damage, and promote genomic instability—a hallmark of cancer progression. On the other hand, ROS can activate signaling pathways that enhance cell survival, proliferation, and migration [18,19,20]. This dual role of ROS is exploited by drug-resistant cells, which increases their antioxidant defenses to survive under the oxidative stress caused by chemotherapy [20,21,22].

Moreover, the metabolic plasticity of cancer cells allows them to switch between different carbon sources depending on their environment. While glucose is the primary fuel for most cancer cells, drug-resistant cells can also rely on alternative carbon sources such as glutamine. Glutamine serves as a critical substrate for replenishing the tricarboxylic acid (TCA) cycle and generating biosynthetic precursors needed for cell growth and survival; this phenomenon is known as glutamine addiction [23,24,25,26,27].

This metabolic flexibility is a survival mechanism and a key contributor to therapy resistance. Resistant cancer cells can adapt to the metabolic stress imposed by chemotherapy, shifting their reliance from glucose to oxidative phosphorylation or glutamine metabolism. We and others have reported that resistant NSCLC cells exhibit reduced glycolytic activity but show increased mitochondrial respiration, making them less sensitive to glycolysis-targeting therapies and more vulnerable to agents that disrupt mitochondrial function [2,28,29].

The metabolic reprogramming seen in drug-resistant cancers underscores the complexity of targeting tumor metabolism as a therapeutic strategy. While glycolysis-targeting drugs have shown promise in the treatment of cancer, their efficacy is limited in resistant tumors that shift toward oxidative metabolism. Understanding these metabolic shifts is critical for developing novel therapies that can target the unique metabolic vulnerabilities of resistant cancer cells.

## 3. Key Metabolic Pathways in Drug Resistance

The metabolic flexibility of cancer cells is one of the key drivers of therapy resistance, allowing them to evade the effects of conventional treatments such as chemotherapy, targeted therapies, and immune checkpoint inhibitors (ICIs). As tumors evolve, they shift their metabolic programs, relying on a variety of pathways that support survival, proliferation, and metastasis under therapeutic stress. Understanding these key pathways is essential for developing strategies to overcome resistance (Table 1).

### 3.1. Glycolysis and Pyruvate Kinase M2 (PKM2)

**Figure 1:**
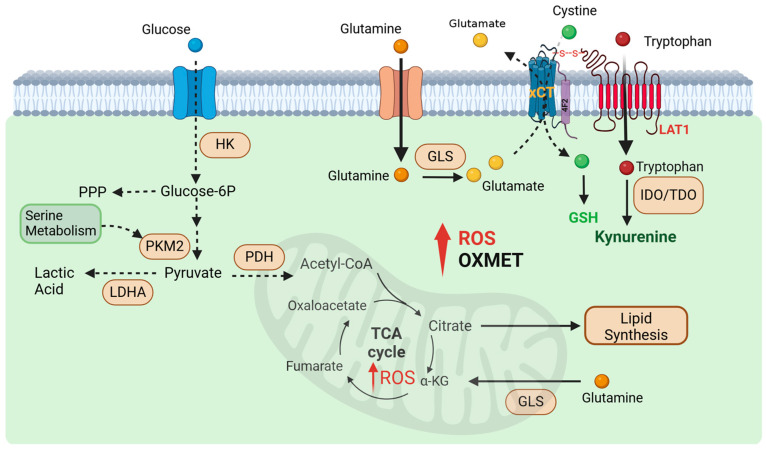
Metabolic reprogramming in cancer cells that represent treatment targets. Resistant tumors are less reliant on glycolysis and show decreased glucose uptake, shifting their metabolic dependency toward amino acid metabolism. They consume large amounts of glutamine, which is hydrolyzed to glutamate to fuel the TCA cycle, support oxidative phosphorylation, and synthesize glutathione—an essential factor for neutralizing high ROS levels via the xCT antiporter. Additionally, resistant tumors display elevated fatty acid synthesis enzymes, reflecting increased lipid biosynthesis. They also engage in tryptophan catabolism, producing kynurenine (KYN), an oncometabolite linked to poor cancer prognosis. The xCT and LAT1 transporters, critical for glutamate release and tryptophan uptake, are interconnected through the 4F2 surface antigen.

One of the most studied metabolic alterations in cancer is the Warburg effect, where cancer cells preferentially use aerobic glycolysis over OXPHOS for ATP production. This metabolic pathway is less efficient regarding energy yield, but it supports the biosynthesis of intermediates necessary for rapid cell division. In drug-resistant cancer cells, however, there is a growing reliance on pyruvate kinase M2 (PKM2)—an isoform of pyruvate kinase that plays a critical role in controlling the balance between glycolysis, the pentose phosphate pathway (PPP), and serine biosynthesis [30,31] (Figure 1).

PKM2 allows cancer cells to maintain flexibility in their metabolic program by enhancing glucose uptake and diversion of glycolytic intermediates to the PPP and serine biosynthesis [31,32,33]. This diversion to PPP supports the production of NADPH, which is critical for neutralizing reactive oxygen species (ROS) and maintaining redox balance [34]. By balancing glycolytic flux and antioxidant defenses, resistant cancer cells can survive the oxidative stress imposed by treatments like cisplatin [35]. Inhibiting PKM2 activity has been shown to induce metabolic stress in resistant cancer cells, making them more susceptible to therapies that target other metabolic pathways. Moreover, PKM2 has several unique allosteric effectors, including serine—an intermediate of de novo purine biosynthesis—that activates PKM2 enzymatic activity [31,36]. A study using a different small-molecule activator of PKM2 demonstrated an increased flux into the serine biosynthesis pathway without raising intracellular serine levels [37]. Consistently, decreased PKM2 activity has been shown to increase glucose flux towards serine production [38].

These findings collectively suggest that modulating PKM2 enzymatic activity can alter the flux of glucose carbon into pathways downstream of pyruvate kinase. Thus, inhibition of PKM2 could lead to the accumulation of glycolytic intermediates and disrupt cancer cell proliferation, highlighting its potential as a therapeutic target [39,40]. A PKM2 clinical trial has shown that inhibiting PKM2 significantly enhances the sensitivity of advanced bladder cancer cells to cisplatin, leading to increased apoptosis and reduced cell proliferation. This allows for the role of PKM2 to be monitored for its effect on drug resistance mechanisms. PKM2 has been seen to be a principally upregulated protein during urothelial tumor formation in the low-grade non-invasive pathway of human BC cell lines and tumors, establishing PKM2 overexpression in both low-grade non-invasive and high-grade invasive human BC. Thus, inhibition of PKM2 decreases tumor formation in bladder cancer [41].

### 3.2. Oxidative Phosphorylation and Mitochondrial ROS

While many tumors initially rely on glycolysis, we have demonstrated that cisplatin-resistant cancer cells undergo a second metabolic shift [1,2]. These resistant cells become more reliant on OXPHOS and exhibit increased mitochondrial activity [29]. As a result, they consume more oxygen which also leads to elevated basal ROS levels. A significant body of literature has highlighted the crucial role of ROS in multiple stages of cancer development, including tumorigenesis and the development of drug resistance [42,43]. Moreover, oxidative phosphorylation (OXPHOS) generates high levels of ATP, which fuels ATP-binding cassette (ABC) transporters like P-glycoprotein (P-gp) [44]. These transporters efflux chemotherapeutic drugs out of cells, reducing intracellular drug concentrations and efficacy.

ROS activate many cell signaling pathways, including nuclear factor kappa-light-chain-enhancer of activated B cells (NFκB), a transcription factor that promotes cell survival, inflammation, and drug resistance. NF-κB can upregulate anti-apoptotic proteins like Bcl-2 and PD-L1 expression, aiding immune evasion [45]. Excessive ROS cause DNA damage, leading to mutations that can activate oncogenic signaling pathways or inactivate tumor suppressor genes [46]. DNA damage response (DDR) mechanisms, such as ATR and ATM activation, also confer resistance by enhancing DNA repair capacity, mitigating the efficacy of DNA-damaging agents like cisplatin.

As previously reported by Trachootham et al. in chronic myeloid leukemia (CML) cells, one can push resistant cells beyond their ROS tolerance limit and ultimately lead to cell death [47]. CR cells already possess higher basal levels of ROS; therefore, they are more susceptible to further ROS induction. This concept is supported by our findings and others which showed that elesclomol, an agent that generates ROS, can selectively kill CR cells while sparing normal cells [28,48]. In a phase II clinical trial involving patients with metastatic melanoma, the combination of elesclomol and paclitaxel was found to extend progression-free survival when compared to just using paclitaxel. Combining elesclomol with standard chemotherapy drugs also leads to a more significant apoptotic response compared to chemotherapy alone [49].

Cancer cells that show increased dependence on OXMET are particularly sensitive to OXPHOS inhibition. Drugs like metformin and its analogs inhibit complex I of the electron transport chain, reduce ROS generation, and activate AMPK; this suppresses mTOR signaling, a pathway important for cancer cell growth [50]. Mito-metformin analogs with longer side chains exhibit more potent inhibition of oxygen consumption and cancer cell proliferation by similarly inducing complex I inhibition and AMPK activation [50,51]. A mito-metformin analog has indicated that it is capable of inhibiting cell proliferation in pancreatic ductal adenocarcinoma cells and proved to be nearly 1000 times more effective than metformin alone [50].

### 3.3. Glutamine Addiction and Anaplerosis

Another key aspect of drug-resistant cancers is glutamine addiction, which helps to strengthen the tricarboxylic acid (TCA) cycle and supports anabolic growth [23,25,52]. Glutamine is converted into glutamate, which feeds into the TCA cycle, supporting the production of ATP and essential biomolecules. In resistant cancer cells, this process is critical for sustaining rapid proliferation and overcoming metabolic stress induced by therapy. The enzyme glutaminase (GLS), which catalyzes the conversion of glutamine to glutamate, is frequently upregulated in drug-resistant tumors [53]. Inhibitors of glutaminase, such as CB-839 (telaglenastat), have shown potential in preclinical models by selectively targeting glutamine-addicted cancer cells. These inhibitors disrupt the TCA cycle and reduce ATP production, thereby inducing apoptosis in resistant cells. Telaglenastat showed no significant side effects in preclinical trials and has gone to full clinical trials [54].

Combination therapies that include these glutaminase inhibitors alongside conventional chemotherapy or immune checkpoint inhibitors are currently ongoing. These trials include nivolumab (an immune checkpoint inhibitor) for melanoma, renal cell carcinoma (RCC), and non-small cell lung cancer (NSCLC) (clinicaltrials.gov ID: NCT02771626); everolimus (an mTOR inhibitor) for RCC (clinicaltrials.gov ID: NCT03163667); palbociclib (a CDK4/6 inhibitor) for KRAS-mutated pancreatic ductal adenocarcinoma (PDAC), NSCLC, and colorectal cancer (CRC) (clinicaltrials.gov ID: NCT03965845); and cabozantinib (a tyrosine kinase inhibitor) for advanced RCC (clinicaltrials.gov ID: NCT03428217).

### 3.4. Lactate Dehydrogenase A (LDHA) and Lactate Production

Lactate production is another hallmark of cancer metabolism, particularly in cells undergoing aerobic glycolysis. The enzyme lactate dehydrogenase A (LDHA) facilitates the conversion of pyruvate to lactate, allowing cancer cells to regenerate NAD^+^ and continue glycolysis. In resistant cancers, increased LDHA expression has been associated with enhanced glycolysis and lactate production, supporting tumor growth and survival in hypoxic conditions [55,56].

Inhibiting LDHA has been shown to reduce lactate production and disrupt the metabolic balance in resistant cells, leading to increased ROS production and cell death [57,58]. We showed that CR cells expressed decreased LDHA protein but higher basal levels of ROS. Subsequently, we also demonstrated that Riluzole, an FDA-approved drug for the treatment of amyotrophic lateral sclerosis (ALS), led to a further decrease in NAD^+^ and LDH expressions and heightened oxidative stress in CR cells [26]. Another clinical trial demonstrated that Riluzole inhibited brain tumor stem-like cell growth. This is due to its inhibition of glucose transporter 3 (GLUT3), as the presence of GLUT3 is indicative of poor prognosis for many cancers, including lung cancer [59].

The LDHA clinical trial has depicted that inhibiting LDHA expression leads to the reduction of cell proliferation, a marked delay in tumor migration, and in vivo tumorigenesis. By inhibiting LDHA, lactate production was shown to decrease, leading to a buildup of pyruvate, thereby decreasing glycolysis and reducing cell proliferation in targeted tumor cells [60,61].

## 4. Kynurenine Pathway as a Therapeutic Target

The KYN pathway is emerging as a key player in cancer metabolism and immune evasion. This pathway, responsible for the catabolism of the amino acid tryptophan (TRP), is exploited by cancer cells to suppress immune surveillance and promote tumor progression. Several enzymes involved in the KYN pathway, including indoleamine 2,3-dioxygenase 1 (IDO1) and tryptophan 2,3-dioxygenase (TDO2), are upregulated in cancer cells and contribute to therapy resistance. By manipulating the KYN pathway, cancer cells can create an immunosuppressive microenvironment, making it an attractive target for therapeutic intervention [62,63].

### 4.1. Tryptophan and System XC-Cystine/Glutamate Antiporter Pump Axis

We found that while uptake of TRP is significantly increased in CR cells, the NAD^+^ levels and QPRT (quinolinate phosphoribosyltransferase) expressions were significantly down-regulated when compared to parental cell counterparts [64]. Hence, resistant cells actively utilize the KYN pathway but do not engage in de novo synthesis of NAD^+^. Instead, KYN is being transported out into the extracellular space [65]. We also reported that CR cells expressed higher levels of the SLC7A11 (xCT) antiporter pump and can be targeted by Riluzole [26]. xCT is a component of a plasma membrane transporter that mediates the cellular uptake of extracellular cystine in exchange for intracellular glutamate and plays a key role in glutathione (GSH) antioxidant synthesis (Figure 1).

Studies showed that the xCT system requires 4F2 heavy chain (4F2hc) and light chain amino acid transporters (LAT1) to be fully functional [66,67]. LAT1 is bound to 4F2hc by a disulfide bond and transports large neutral amino acids like tryptophan (TRP) [66,67,68] (Figure 1). It has been shown that resistant cells not only expressed increased xCT but also increased LAT1, both crucial components for transport [26,64,69,70]. Thus, targeting the xCT pump with Riluzole may influence TRP transporters, subsequently reducing KYN production. This mechanism suggests that Riluzole could be a promising candidate for combination therapy with immune checkpoint inhibitors to enhance anti-tumor efficacy.

### 4.2. Tryptophan Catabolism and Immune Suppression

The primary role of the KYN pathway is to break down tryptophan into several metabolites, including kynurenine, which has immunosuppressive properties. In cancer, overexpression of IDO1 and TDO2 leads to increased tryptophan degradation, depleting local tryptophan levels and resulting in T cell anergy and immune escape [63,71]. The accumulation of kynurenine in the tumor microenvironment suppresses the activity of effector T cells while promoting the expansion of regulatory T cells (Tregs), further enhancing immune suppression [62,63].

Additionally, KYN activates the aryl hydrocarbon receptor (AhR), which drives immune suppression and facilitates tumor growth and metastasis. The activation of AhR by KYN also promotes the differentiation of myeloid-derived suppressor cells (MDSCs), further contributing to the immunosuppressive environment [72,73]. These effects collectively help cancer cells evade immune detection and destruction.

### 4.3. IDO1 and TDO2 as Therapeutic Targets

The enzymes IDO1 and TDO2 are considered key therapeutic targets in the KYN pathway due to their role in immune suppression and tumor progression. Inhibition of these enzymes has been shown to restore anti-tumor immunity in preclinical models by increasing local tryptophan levels and reducing the immunosuppressive effects of KYN. Several IDO1 inhibitors, such as epacadostat, have been developed and tested with promising results when combined with immune checkpoint inhibitors [74,75,76]. Most researchers agree that IDO1 inhibition may synergize well with ICIs, potentially leading to improved anti-tumor outcomes [77].

In fact, IDO1 inhibitors had already gone to Phase 3 trial (Keynote-252/ECHO-301) with epacadostat in combination with ICIs for melanoma [78]. However, the trial was met with early termination due to no discernible increase in benefits with epacadostat. There are several key factors contributing to the failure of IDO1 inhibition trials, including the failure to select the appropriate patient population; it is crucial to first determine whether the tumor relies on the KYN pathway as its primary metabolic dependency before selecting it for the study.

### 4.4. Compensatory Effect from TDO2

While previous publications on IDO inhibition trials have highlighted various limitations such as the adequacy of IDO1 inhibition within the tumor, proper dosage, and drug exposure, one of our findings that began to address the lack of efficacy of IDO inhibition was our demonstration of the compensatory role of TDO2 in overcoming the blockade of IDO1 [79]. In this study, we showed that inhibition of IDO1, either by inhibitors or CRISPR knockout, led to a significant increase in TDO2 expression. This data strongly supports the presence of a compensatory mechanism wherein NSCLC-CR tumors activate IDO1 and/or TDO2 to overcome single enzyme pharmacological blockade as therapy.

The potential synergy between KYN pathway inhibitors and immune checkpoint blockade has been a major focus of many studies. Combining IDO1/TDO2 inhibitors with PD-1/PD-L1 inhibitors could be one of the most promising strategies for overcoming immune suppression by restoring T cell activity and reversing the immunosuppressive tumor microenvironment. In addition to IDO1 and TDO2 inhibitors, research is exploring the role of AhR antagonists as a means of blocking kynurenine-mediated immune suppression. Targeting AhR could prevent the downstream effects of kynurenine accumulation, including the expansion of Tregs and MDSCs, thereby restoring a more favorable immune environment for tumor destruction [80,81].

### 4.5. Current Challenges and Future Directions

While targeting the KYN pathway offers a novel therapeutic avenue, challenges remain in fully understanding its role in cancer biology. Not all cancers exhibit high levels of IDO1 or TDO2 expression, and the effectiveness of KYN pathway inhibitors may vary depending on tumor type and microenvironment. Additionally, resistance mechanisms to single IDO1 inhibitors have been observed [82,83], suggesting that further research is needed to optimize the use of these therapies.

Future directions in targeting the kynurenine pathway include the development of dual inhibitors that target both IDO1 and TDO2, as well as the investigation of combination therapies that include metabolic inhibitors, ROS inducers, and other agents that disrupt cancer metabolism. These strategies may offer new ways to overcome the immune evasion tactics employed by resistant tumors.

## 5. Immune Checkpoints and Metabolism in Resistance

Cancer cells’ ability to evade immune detection and destruction is a hallmark of tumor progression and therapy resistance. Immune checkpoint molecules like PD-L1 (Programmed Death-Ligand 1) and PD-1 (Programmed Death-1) have been shown to play a crucial role in facilitating immune escape in cancer. These immune checkpoints, when activated, inhibit the anti-tumor activity of T cells, allowing tumors to thrive [84,85,86]. In recent years, a growing body of evidence has emerged linking metabolic reprogramming in cancer cells with immune evasion mechanisms, particularly through the regulation of PD-L1 expression [87,88].

### 5.1. ROS and PD-L1 Expression Axis

Metabolic alterations in cancer cells are intricately connected to immune evasion strategies. One of the primary metabolic shifts observed in resistant cancer cells is the upregulation of PD-L1, which is associated with immune checkpoint resistance. This upregulation is often triggered by metabolic stressors such as hypoxia and ROS, both of which are prevalent in the tumor microenvironment of drug-resistant cancers [89,90]. Hypoxia in the TME stabilizes HIF1α (hypoxia-inducible factor 1-alpha), a transcription factor that promotes the expression of PD-L1. HIF1α directly binds to hypoxia-response elements (HREs) in the PD-L1 promoter, driving its transcription [91]. As for ROS, it can act as signaling molecules by activating NFκB pathways [45,90]. This dual activation promotes a more robust expression of PD-L1. The synergistic effect of these pathways exacerbates immune evasion and creates a microenvironment conducive to resistance mechanisms.

Studies have shown that increased ROS levels and shifts toward oxidative metabolism in resistant cells promote epithelial–mesenchymal transition (EMT), a process associated with increased tumor aggressiveness and metastasis. While EMT is a well-characterized consequence of ROS, its role in drug resistance extends beyond motility and invasion. EMT-associated transcription factors, such as Snail and Twist, have been implicated in upregulating PD-L1 [92,93]. The induction of EMT facilitates immune evasion by increasing PD-L1 expression on the surface of cancer cells, thereby inhibiting T cell-mediated killing [92,94]. In platinum-resistant lung cancer cells, metabolic reprogramming through OXPHOS leads to elevated ROS production, which triggers EMT and enhances PD-L1 expression [64,93,95]. This process creates a feedback loop in which metabolic adaptation not only drives therapy resistance but also enhances immune evasion, making these tumors more difficult to treat with conventional therapies alone.

Integrating ROS-induced EMT with the HIF1α and NFκB pathways highlights a networked regulation of PD-L1, reinforcing its role in immune escape and resistance.

### 5.2. Interaction Between Metabolic Pathways and Immune Checkpoints

#### 5.2.1. Aerobic Glycolysis and Immune Evasion

The relationship between cancer cell metabolism and immune checkpoints is bidirectional. Not only does metabolic reprogramming influence immune checkpoint expression, but immune checkpoints themselves can modulate metabolic pathways within the tumor microenvironment. The activation of PD-1 on T cells can lead to metabolic exhaustion, impairing the T cells’ ability to function effectively against tumor cells; this exhaustion is characterized by reduced glycolysis and mitochondrial dysfunction in T cells [96]. At the same time, tumors with high PD-L1 expression can exhibit enhanced glycolysis and lactate production, which contribute to an immunosuppressive microenvironment. Driven by increased glycolysis in cancer cells, high lactate levels can inhibit T cell function by reducing the local pH, creating conditions unfavorable for an effective immune response [97,98]. Acidic pH and lactate can promote PD-L1 expression through signaling pathways such as STAT3 and NFκB, reinforcing immune evasion [99].

#### 5.2.2. Nucleotide Metabolism and Immune Evasion

This pathway is essential for supporting the rapid proliferation of cancer cells. Dysregulation of this pathway has been increasingly linked to immune evasion, including the upregulation of PD-L1. Both the de novo synthesis and salvage pathways for purines and pyrimidines, which are critical for DNA and RNA synthesis as well as various signaling functions, play a role in this process.

Elevated nucleotide synthesis activates the mTOR pathway, a key regulator of cellular growth and metabolism. mTOR signaling, through downstream effectors such as STAT3 and HIF1α, can directly promote PD-L1 transcription [100,101]. Furthermore, an imbalance in nucleotide pools can increase biosynthetic activity, triggering oxidative stress and DNA damage responses. These, in turn, activate immune-modulating pathways like the cGAS-STING pathway. Interestingly, this pathway can paradoxically enhance PD-L1 expression as a tumor defense mechanism or make tumors more susceptible to immunotherapy when paired with checkpoint inhibitors.

Increased pyrimidine synthesis has also been associated with immune evasion and heightened PD-L1 expression via the activation of oncogenic signaling pathways such as tyrosine kinase [102,103]. This highlights the broader impact of metabolic reprogramming on tumor-immune interactions, underscoring how the metabolic demands of nucleotide production can integrate with immune evasion strategies to promote tumor survival and progression.

#### 5.2.3. Amino Acid Metabolism and Immune Evasion

Amino acid metabolism plays a crucial role in supporting tumor growth and modulating the immune microenvironment. The dysregulated metabolism of key amino acids—such as tryptophan, glutamine, and arginine—has been linked to the upregulation of PD-L1 and subsequent immune evasion.

Kynurenine pathway: KYN acts as an immunosuppressive metabolite and has been shown to upregulate PD-L1 expression through activation of the aryl hydrocarbon receptor (AhR) [104,105]. KYN binds to AhR, a transcription factor that promotes the expression of immunosuppressive genes, including PD-L1. This pathway also suppresses T cell proliferation and promotes regulatory T cell differentiation, enhancing immune evasion.

Glutamine pathway: Studies have shown that limiting glutamine availability can lead to increased expression of PD-L1 in tumor cells [106]. Glutamine deprivation in the culture medium upregulated the expression of PD-L1 on renal cancer cells via the EGFR/ERK/C-Jun pathway [106]. Decreased glutamine levels can lead to a reduction in GSH levels, which in turn inhibits sarcoplasmic reticulum Ca^2+^-ATPase (SERCA) activation and increases cytosolic Ca^2+^ levels and CaMKII phosphorylation. This further activates the downstream nuclear factor-kappa B (NF-κB) signaling pathway to promote the expression of PD-L1 [107].

Arginine pathway: We and others have shown that targeting arginine is a promising approach for the treatment of various malignancies [108,109,110]. Moreover, arginine is a critical amino acid involved in the production of nitric oxide (NO) and polyamines, which play roles in cell signaling, proliferation, and immune modulation. Overexpression of arginase in the tumor microenvironment depletes arginine, impairing T cell function and creating an immunosuppressive milieu [111,112]. Arginase activity can also enhance PD-L1 expression via increased polyamine biosynthesis and the activation of transcription factors such as STAT3 [113,114].

### 5.3. Targeting Metabolic Vulnerabilities and Immune Checkpoints

The interplay between metabolism and immune checkpoints presents an opportunity for combination therapies that target both aspects of tumor biology. By disrupting the metabolic pathways that support immune evasion, it is possible to enhance the effectiveness of immune checkpoint inhibitors. For instance, combining ROS-inducing agents with PD-1 or PD-L1 inhibitors has shown promise in preclinical models, as the increased oxidative stress in resistant cancer cells can sensitize them to immune-mediated cell death [115].

Limiting glutamine availability can enhance PD-L1 expression [106,107]. However, when combined with glutamine inhibition with anti-PD-L1 therapy, it enhances the antitumor efficacy of T cells both in vitro and in vivo [107]. It is noteworthy that higher PD-L1 expression is associated with improved clinical outcomes for patients undergoing treatment with ICIs, particularly in the context of NSCLC. Thus, it is possible that targeting glutamine metabolism, which is upregulated in resistant cancer cells, can enhance the efficacy of immune checkpoint therapies.

High levels of circulating lactic acid/LDHA expression are one of the major causes of primary resistance to anti-PD-L1 immunotherapy [116]. Studies have proven that low levels of LDHA were associated with better anti-PD-1 antibody therapeutic responses in patients with melanoma [117].

Blocking LDHA in combination with anti-PD increases the infiltration of CD8+ cytotoxic T cells and natural killer (NK) cells, as well as increases the production of interferon-γ (IFN-γ) and granzyme B in vivo [118].

### 5.4. Clinical Implications of Combining Immune Checkpoint and Metabolism-Targeting Therapies

The combination of immune checkpoint inhibitors with metabolism-targeting agents is an emerging strategy to overcome therapy resistance. Clinical trials are already underway investigating the efficacy of PD-1/PD-L1 inhibitors in combination with agents that disrupt cancer metabolism, such as ROS inducers, OXPHOS inhibitors, and glutaminase inhibitors. Early results suggest that these combinations can enhance the immune response by weakening the metabolic defenses of resistant tumors and restoring T cell activity [119,120].

Furthermore, the epigenetic regulation of PD-L1 through metabolic pathways has been identified as a novel area of research. Studies suggest that certain metabolic intermediates, such as those produced by the kynurenine (KYN) pathway, may regulate the expression of PD-L1, presenting new opportunities for targeted intervention. The inhibition of rate-limiting enzymes like IDO1 and/or TDO2 in the KYN pathway has already shown potential in preclinical models, providing a foundation for combining such inhibitors with immune checkpoint therapies [121,122,123].

## 6. Conclusions and Future Directions

The field of cancer therapy has seen remarkable advancements with the development of targeted therapies and immune checkpoint inhibitors, yet drug resistance remains a major obstacle. As understanding of cancer metabolism grows, it is becoming increasingly clear that targeting the metabolic weaknesses of drug-resistant tumors can provide new avenues for overcoming resistance (Box 1).

Box 1 **Key Findings.**

Metabolic reprogramming in cancer cells, including shifts from glycolysis to OXPHOS and the reliance on alternative carbon sources (ex. glutamine), allows tumors to survive and resist therapies such as chemotherapy and immune checkpoint blockade.Elevated reactive oxygen species (ROS) and lactate production contribute to immune evasion, creating an immunosuppressive tumor microenvironment that reduces the efficacy of immune-based therapies.The kynurenine pathway plays a crucial role in immune suppression through the activity of enzymes like IDO1, which further inhibits anti-tumor immune responses.Combining metabolic inhibitors (such as glutaminase inhibitors, LDHA inhibitors, and OXPHOS inhibitors) with immune checkpoint inhibitors presents a promising strategy to target both the metabolic and immune escape mechanisms of resistant tumors.

The next frontier in cancer therapy lies in integrating metabolic inhibitors with other targeted therapies to make treatments the most effective and personalized for each patient. Establishing reliable biomarkers, essential for selecting patients who can benefit most from these combination therapies, and expanding the understanding of tumor metabolism across cancer types are critical future steps in opening new doors for therapeutic interventions [124,125] (Box 2).

Box 2 **Future Research and Challenges.**

**Challenges****Rationales**Cancer cells exhibit diverse metabolic profiles even within the same tumor, greatly complicating the ability to target specific metabolic pathways across all patients.Drugs that simultaneously inhibit multiple metabolic pathways, such as amino acid synthesis pathway and OXMET, could potentially address this metabolic flexibility and force tumors into an energy crisis.As more is understood about the interaction between metabolism and immune evasion, targeting immune suppressive cells, like Tregs or MDSCs, in combination with metabolic inhibitors could further enhance anti-tumor responses.Since many metabolic inhibitors target fundamental cellular processes, concerns about toxicity to normal tissues must be addressed.Biomarkers can be used to identify patients who are most likely to benefit from specific metabolic inhibitors based on their tumors’ metabolic profiles.Developing more methods to profile the metabolic dependencies of individual tumors will enable more personalized approaches, ensuring that therapies target the most critical metabolic vulnerabilities in each patient.

Ultimately, the combination of metabolic reprogramming inhibitors and immune therapies offers great potential to not only overcome drug resistance but also improve overall patient outcomes. The future of cancer therapy will likely require a multifaceted approach that disrupts the complex metabolic and immune networks that cancer cells rely on to thrive.

## Figures and Tables

**Table 1:** Drugs targeting tumor metabolism.

Drug	Metabolism Target	Target Cancer Disease	Clinical Trials	Status
Elesclomol	Disrupting mitochondrial metabolism	Lung, ovarian, prostate	NCT00088088, NCT00888615, NCT00808418	Showed increased cancer cell death and increased survival times when used with paclitaxel
Epacadostat	IDO1 inhibitor	Breast, lung, melanoma, prostate	NCT02178722, NCT02862457, NCT02752074, NCT03493945	Tested in combination with immune checkpoint inhibitors, stopped in Phase III due to failure to show significant benefit
Everolimus	mTOR inhibitor	Brain, breast, lung, neuroendocrine	NCT01062399, NCT02229136, NCT01470209, NCT03070301	Improved PFS when used in telaglenastat in the 2017 Phase II trial
Metformin	ETC complex I inhibitor	Breast, colon, ovarian, pancreatic, prostate	NCT01589367, NCT03359681, NCT02312661, NCT01210911, NCT01796028	Mito-Met has proven to enhance anti-cancer activity by slowing cancer cell proliferation and oxygen consumption
Telaglenastat	GLS inhibitor	Breast, prostate, renal	NCT03057600, NCT03163667, NCT04824937	Currently, in full clinical trials; preclinical trials had no significant side effects
Riluzole	Glutamate release inhibitor	Breast, brain, melanoma	NCT00903214, NCT01018836, NCT00866840	Had success in killing CR cells in vivo and in vitro, is currently on the market

## Data Availability

No new data were created or analyzed in this study. Data sharing is not applicable to this article.

## Abbreviations

AhR	aryl hydrocarbon receptor
ALS	amyotrophic lateral sclerosis
BC	breast cancer
CML	chronic myeloid leukemia
CR	cisplatin-resistant
EGFR	epidermal growth factor receptor
EMT	epithelial-mesenchymal transition
GLS	glutaminase
GLUT3	glucose transporter 3
GSH	glutathione
HIF1α	hypoxia-inducible factor 1-alpha
ICI	immune checkpoint inhibitor
IDO1	indoleamine 2,3-dioxygenase-1
KYN	kynurenine
LAT1	light chain amino acid transporters
LDHA	lactate dehydrogenase A
MDSC	myeloid-derived suppressor cells
NO	nitric oxide
OXMET	oxidative metabolism
OXPHOS	oxidative phosphorylation
PD-L1	programmed death-ligand 1
PD-1	programmed death 1
PK	pyruvate kinase
PPP	pentose phosphate pathway
QPRT	quinolinate phosphoribosyltransferase
ROS	reactive oxygen species
NSCLC	non-small cell lung cancer
TCA	tricarboxylic acid
TDO2	tryptophan 2,3-dioxygenase-2
TRP	tryptophan
